# Emerging pan-resistance in *Trichosporon* species: a case report

**DOI:** 10.1186/s12879-016-1477-3

**Published:** 2016-04-14

**Authors:** Claudy Oliveira dos Santos, Jan G. Zijlstra, Robert J. Porte, Greetje A. Kampinga, Anne D. van Diepeningen, Bhanu Sinha, Erik Bathoorn

**Affiliations:** Department of Medical Microbiology (EB 80), University Medical Center Groningen, P.O. Box 30 001, 9700 RB Groningen, The Netherlands; Department of Critical Care, University of Groningen, University Medical Center Groningen, Groningen, The Netherlands; Department of Hepatobiliary Surgery, University of Groningen, University Medical Center Groningen, Groningen, The Netherlands; CBS-KNAW Fungal Biodiversity Center, Utrecht, The Netherlands

**Keywords:** *Trichosporon dermatis*, Disseminated trichosporonosis, Invasive fungal disease, Antifungal stewardship, Liver transplant

## Abstract

**Background:**

*Trichosporon* species are ubiquitously spread and known to be part of the normal human flora of the skin and gastrointestinal tract. *Trichosporon* spp. normally cause superficial infections. However, in the past decade *Trichosporon* spp*.* are emerging as opportunistic agents of invasive fungal infections, particularly in severely immunocompromised patients. Clinical isolates are usually sensitive to triazoles, but strains resistant to multiple triazoles have been reported.

**Case presentation:**

We report a high-level pan-azole resistant *Trichosporon dermatis* isolate causing an invasive cholangitis in a patient after liver re-transplantation. This infection occurred despite of fluconazole and low dose amphotericin B prophylaxis, and treatment with combined liposomal amphotericin B and voriconazole failed.

**Conclusion:**

This case and recent reports in literature show that not only bacteria are evolving towards pan-resistance, but also pathogenic yeasts. Prudent use of antifungals is important to withstand emerging antifungal resistance.

## Background

The genus *Trichosporon* with its yeast-like anamorphic cells belongs to the phylum of the Basidiomycota [1]. *Trichosporon* spp*.* are present ubiquitously and are known to be part of the normal human flora of the skin and gastrointestinal tract. In otherwise healthy individuals *Trichosporon* spp. may cause infections like white piedra or skin infections. However, in the past decade, *Trichosporon* spp. are emerging as opportunistic agents of invasive fungal infections, particularly in severely immunocompromised patients such as hematological patients and solid organ transplant recipients [2–4]. These patients commonly receive antifungal prophylaxis to prevent invasive fungal infections. Prolonged or recurrent usage of antifungal agents may select for intrinsically resistant fungi, or cause acquisition of resistance in wild-type susceptible fungal species.

Here we present the first case of treatment failure of cholangitis caused by a pan-azole resistant *Trichosporon dermatis*, and we review the literature for voriconazole resistance in *Trichosporon* spp*.*

## Case presentation

A 48-year-old male patient was admitted to our hospital with spontaneous bacterial peritonitis (SBP) and hepatic encephalopathy. He had received a liver transplant 20 years ago for cryptogenic liver cirrhosis. Recently, he had suffered from progressive chronic transplant failure for which he had been repeatedly admitted due to decompensated liver cirrhosis. Other relevant diseases of the patient’s medical history are colitis ulcerosa and diabetes mellitus type II.

**Fig. 1:**
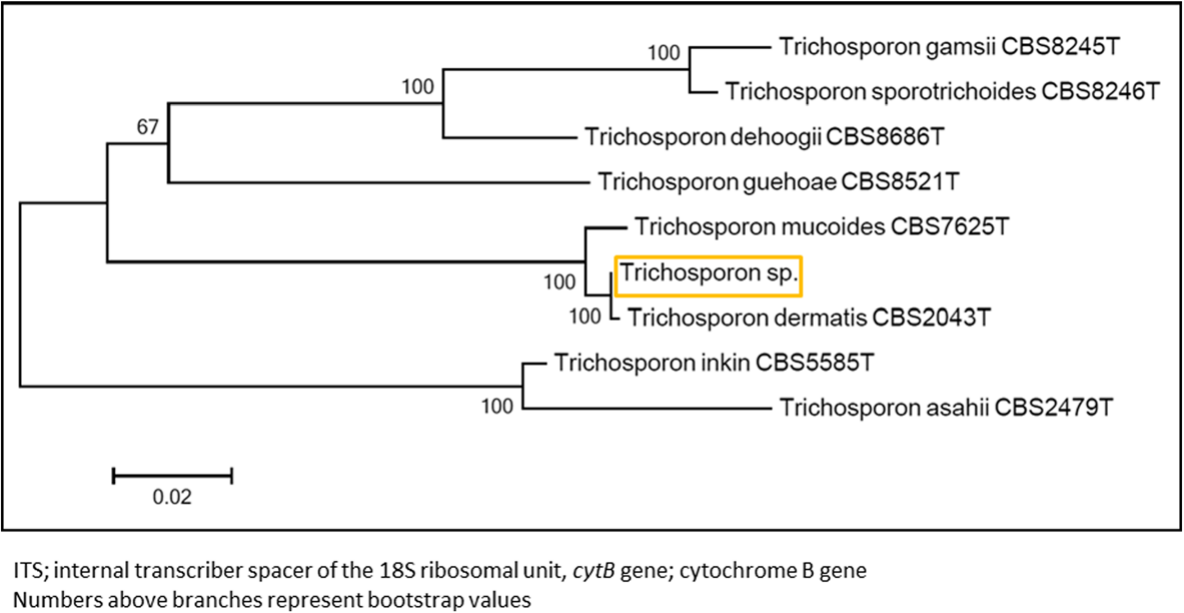
Rooted phylogenetic tree of the *Trichosporon* isolate based on confidently aligned ITS and *cytB* sequences, obtained by maximum parsimony cluster analysis and 1000 bootstrap simulations

The patient was treated with ceftriaxone (2000 mg q.d. IV) for SBP caused by *E. coli*. After two weeks, the patient became febrile again due to recurrent SBP, for which he empirically received meropenem (1000 mg b.i.d. IV), later switched to piperacillin/tazobactam (4000/500 mg t.i.d. IV). These therapies lead to a clinical and biochemical improvement. He became well enough to be listed for liver re-transplantation, which was performed 2 weeks later from a heart beating donor. The preoperative modal for end-stage liver disease (MELD) score was 40 and the portal vein was thrombosed. The transplant procedure was characterized by a very difficult hepatectomy due to massive adhesions, portal hypertension and thickened peritoneum. An attempt to perform thrombectomy of the portal vein was unsuccessful and the donor portal vein had to be anastomosed with a large dilated side branch of the superior mesenteric vein using a iliac vein interposition graft. Because of massive ongoing blood loss the abdomen had to be packed with gauzes. The immediate postoperative course was complicated by primary non-function of the liver graft and portal vein thrombosis, requiring a re-re-transplant two days later. After this re-re-transplant the abdominal wall could not be closed due to massive distension of the viscera. The abdomen was temporarily closed with a silastic mesh. The patient was treated with prophylactic low dose amphotericin B (0.3 mg/kg q.d. IV), fluconazole (100 mg q.d. IV) and piperacillin/tazobactam (4000/500 mg t.i.d. IV) as part of the post-transplantation protocol and selective decontamination of the digestive tract (SDD) regime on the intensive care unit (ICU) consisting of 2 % polymyxin B, 2 % tobramycin and 2 % amphotericin B in a suspension (0.5 gram q.d.s.) and oral paste in the mouth. On day seven after the second transplantation the patient clinically deteriorated due to an abdominal compartment syndrome. Cultures taken from the abdominal drains 5 days later yielded *Trichosoporon* species. Additional samplings from the 4 abdominal drains in situ were also positive for *Trichosporon*. Species determination by Matrix Assisted Laser Desorption Ionization-Time of Flight mass spectrometry (MALDI-TOF MS, Bruker) showed *T. mucoides* with a score of 2.17. Molecular sequencing of the internal transcriber spacer (ITS) and cytochrome B gene (*cytB*) by CBS-KNAW Fungal Biodiversity Center, Utrecht, The Netherlands (Fig. 1), conclusively identified the isolate as *T. dermatis* (deposited in Genbank under accession numbers KT597976 and KT597975, respectively and Treebase under submission number S18915). The isolate has been deposited in the CBS-KNAW Fungal Biodiversity Center under catalog number CBS14086.

Table 1 shows the MICs for all antifungal agents tested. Amphotericin B prophylaxis was switched to liposomal amphotericin B (5 mg/kg q.d. IV) and fluconazole was switched to voriconazole (200 mg b.i.d. IV). Tapering of immunosuppressive therapy (hydrocortisone 4 mg/h continuous IV) was not deemed feasible. The remaining anti-rejection therapy consisted of basiliximab 20 mg on day 0 and day 4 after transplantation.

**Table 1:** MICs of various antifungal agents against 11 clinical *Trichosporon dermatis* isolates

*T. dermatis* strains tested	MIC (mg/liter)														Method	Reference
	Amphotericin B^a^		Flucytosine		Fluconazole		Itraconazole		Voriconazole		Posaconazole		Caspofungin			
	GM	Range	GM	Range	GM	Range	GM	Range	GM	Range	GM	Range	GM	Range		
1	0.25		>64		>256		>16		>16		>8		>16		Sensititre^b^	Our study
8	0.24	0.015-16.0	58.6	16.0-128.0	12.3	1.0-128.0	0.58	0.06-8.0	0.48	0.06-16.0	ND		ND		Eucast	Rodriguez-Tudela et al 2005 [7]
1	0.5		8				0.06		0.06		ND		16		CLSI	Chagas-Neto et al 2009 [2]
1	0.13		ND		2		0.06		0.06		0.015		ND		Eucast	Taverna et al. 2014 [16]

*GM* geometrical mean, *MIC* minimal inhibitory concentration, *ND* not done

^a^Effectiveness of amphotericin B is limited in *Trichosporon* infections [12]

^b^Susceptibility was tested using a commercial microdilution test (Yeast Sensititre OneTM) by the national reference center (Radboud University Medical Center, Nijmegen, The Netherlands)

Three days later the abdomen was re-explored and the silastic mesh was removed. Cultures from fibrin clots and fluid collections behind the vena cava, suprahepatic material, gallbladder fluid and pleural fluid all yielded pure cultures of *T. dermatis*, which lead to the diagnosis invasive fungal cholangitis. After 1 week of treatment, *T. dermatis* was still cultured from drain fluid. The patient died on the 29^th^ day after the last liver transplantation due to multi-organ failure with persistent trichosporonosis.

## Conclusion

Our *T. dermatis* isolate was highly resistant to all triazoles, echinocandins, and 5-flucytosine. The patient had been treated with several courses of broad-spectrum antibiotics and antifungal agents for abdominal infections in recent history, and received antifungal prophylaxis with low dose fluconazole and low dose amphotericin B when the infection occurred. He also received SDD. The antimicrobial pressure with broad-spectrum antibacterial drugs (reduced competition) and relatively low dose antifungals has most likely contributed to the selection of this highly resistant isolate.

Wild-type *Trichosporon* spp*.* are susceptible to triazoles, that target the lanosterol 14 alpha-demethylase of the ergosterol pathway. From 2003, fluconazole resistance has increasingly been reported [5–7]. Voriconazole is the most effective antifungal agent against *Trichosporon* spp*.* and is recommended as treatment for trichosporonosis [8]. However, from 2010 onward the first sporadic cases of *Trichosporon* spp*.* resistant to voriconazole were reported [9,10], and a recent study from Greece reported that 38 % of *Trichosporon* isolates had a MIC ≥ 2 mg/L for voriconazole [11]. Most of these strains were susceptible to at least one other triazole, and treatment failures were not described in this study. An overview of susceptibilities of *T. dermatis* described in literature is presented in Table 1. This shows that *T. dermatis* are generally susceptible to voriconazole and itraconazole, and underlines the exceptional level of resistance of our isolate.

Invasive trichosporonosis is a life-threatening condition and optimization of antifungal therapy in an early stage of infection is essential. A provisional susceptibility pattern based on Etests® indicated resistance to fluconazole, with a low MIC for voriconazole (0.094 mg/l).

Based on this, we started treatment with both voriconazole and liposomal amphotericin B. The effectiveness of amphotericin B is very low, probably due to its poor killing activity against *Trichosporon* spp. [5,12]. Nonetheless, it is the only treatment option left in case of high-level resistance to triazoles, since *Trichosporon* spp. are intrinsically resistant to echinocandins and flucytosine [8].

Voriconazole resistance was later confirmed by micro-broth dilution method by the national reference center (Radboud University Medical Center, Nijmegen, the Netherlands). The combination of long standing immune suppression, a difficult re-transplant complicated by primary non-function of the graft and infection with a multi-resistant isolate resulted in failure of treatment.

Prudent use of antifungals is important to withstand emerging antifungal resistance. Effectiveness of fluconazole or low dose amphotericin B as antifungal prophylaxis in high-risk liver transplant patients in prevention of invasive candidiasis is evidence-based, and recommended by Infectious Diseases Society of America (IDSA) guidelines [13]. Prophylaxis prevents morbidity and mortality caused by *Candida albicans* [14,15]. From an antifungal stewardship point of view, fluconazole is preferred for its narrow spectrum, and amphotericin B could be reserved for those patients colonized with fluconazole resistant yeasts. Antifungal prophylaxis for at least 7-14 days postoperative in high-risk patients, and during ICU stay, is recommended [13].

Correct species identification is important for epidemiologic reasons, species-specific virulence and resistance characteristics. For *T. dermatis* bi- or multi-locus sequence analysis with e.g. ITS and *cytB* allows unambiguous identification. Misidentification of *T. dermatis* as *T. mucoides* occurs, but is most likely when only biochemical tests are performed [7]. Next to *T. asahii* (74 %), *T. dermatis* is the second most reported species (12 %) causing invasive infections, but *T. mucoides* is rarely involved in invasive infections [3].

We conclude that our study shows that not only bacteria are evolving towards pan-resistance, but also pathogenic yeast species belonging to *Trichosporon*. Antifungal resistance is a serious threat for health care of immunocompromised patients.

## Consent

Written informed consent was obtained from the patients’ next of kin (wife), due to the fact that the patient himself was not able to sign himself, for publication of this Case Report and any accompanying images. The consent was obtained five days before his death.

### Availability of data and materials

The phylogenetic data has been deposited in the databases Genbank and Treebase and are available under accession numbers KT597976 and KT597975 for Genbank, and under accession URL: http://purl.org/phylo/treebase/phylows/study/TB2:S18915 and submission number 18915 for Treebase.

## Abbreviations

b.i.d.: twice daily (bis in die)
cytB: cytochrome B gene
ICU: intensive care unit
IDSA: infectious diseases society of America
ITS: internal transcriber spacer
IV: intraveneous
kg: kilogram
MALDI-TOF MS: matrix assisted laser desorption ionization-time of flight mass spectrometry
MELD: modal for end-stage liver disease
mg: milligram
MIC: minimal inhibition concentration
q.d.: daily (quaque die)
q.d.s.: four times a day (quarter die sumendus)
SBP: spontaneous bacterial peritonitis
SDD: selective decontamination of the digestive tract
t.i.d.: three times a day (ter in die)
