# Erratum to: ‘Emerging pan-resistance in *Trichosporon* species: a case report’

**DOI:** 10.1186/s12879-016-1621-0

**Published:** 2016-06-11

**Authors:** Claudy Oliveira dos Santos, Jan G. Zijlstra, Robert J. Porte, Greetje A. Kampinga, Anne D. van Diepeningen, Bhanu Sinha, Erik Bathoorn

**Affiliations:** Department of Medical Microbiology, University of Groningen, University Medical Center Groningen, Groningen, The Netherlands; Department of Critical Care, University of Groningen, University Medical Center Groningen, Groningen, The Netherlands; Department of Hepatobiliary Surgery, University of Groningen, University Medical Center Groningen, Groningen, The Netherlands; CBS-KNAW Fungal Biodiversity Center, Utrecht, The Netherlands

## Erratum

Unfortunately, the original version of this article [1] contained an error. The first affiliation which is also that of the corresponding author was not included correctly and had the wrong address. Below is the correct presentation of this affiliation.

Department of Medical Microbiology, University of Groningen, University Medical Center Groningen, Groningen, The Netherlands.
